# Mesenchymal Stromal Cell-Based Therapy: New Perspectives and Challenges

**DOI:** 10.3390/jcm8050626

**Published:** 2019-05-08

**Authors:** Mehdi Najar, Fatima Bouhtit, Rahma Melki, Hassan Afif, Abdellah Hamal, Hassan Fahmi, Makram Merimi, Laurence Lagneaux

**Affiliations:** 1 Osteoarthritis Research Unit, University of Montreal Hospital Research Center (CRCHUM), and Department of Medicine, University of Montreal, Montreal, QC H2X 0A9, Canada; hassafif@gmail.com (H.A.); h.fahmi@umontreal.ca (H.F.); 2 Laboratory of Physiology, Genetics and Ethnopharmacology, Faculty of Sciences, University Mohammed Premier, Oujda 60000, Morocco; bouhtitfatima@gmail.com (F.B.); r.melki@ump.ac.ma (R.M.); abdhamal@gmail.com (A.H.); macmerimi@gmail.com (M.M.); 3 Laboratory of Experimental Hematology, Jules Bordet Institute, Université Libre de Bruxelles, 1000 Bruxelles, Belgium; 4 Laboratory of Clinical Cell Therapy, Jules Bordet Institute, Université Libre de Bruxelles, 1070 Bruxelles, Belgium; laurence.lagneaux@bordet.be

**Keywords:** immunity, therapy, mesenchymal stromal cells, immunomodulation, licensing, extracellular vesicles

## Abstract

Stem cells have been the focus of intense research opening up new possibilities for the treatment of various diseases. Mesenchymal stromal cells (MSCs) are multipotent cells with relevant immunomodulatory properties and are thus considered as a promising new strategy for immune disease management. To enhance their efficiency, several issues related to both MSC biology and functions are needed to be identified and, most importantly, well clarified. The sources from which MSCs are isolated are diverse and might affect their properties. Both clinicians and scientists need to handle a phenotypic-characterized population of MSCs, particularly regarding their immunological profile. Moreover, it is now recognized that the tissue-reparative effects of MSCs are based on their immunomodulatory functions that are activated following a priming/licensing step. Thus, finding the best ways to pre-conditionate MSCs before their injection will strengthen their activity potential. Finally, soluble elements derived from MSC-secretome, including extracellular vesicles (EVs), have been proposed as a cell-free alternative tool for therapeutic medicine. Collectively, these features have to be considered and developed to ensure the efficiency and safety of MSC-based therapy. By participating to this Special Issue “Mesenchymal Stem/Stromal Cells in Immunity and Disease”, your valuable contribution will certainly enrich the content and discussion related to the thematic of MSCs.

Cell-based therapy is being increasingly considered an efficacious and safe option for different therapeutic applications. Due to unique properties, the use of stem cells in new medicinal therapies includes treatment of different conditions [1]. One of the main goals of regenerative and personalized medicine is the development of cellular therapies free of side effects and devoid of ethical concerns. However, clinical application of stem cells raises numerous ethical and safety concerns. In particular, the destruction of a human embryo is a major factor that may have limited the development of human embryonic stem cell (hESC)-based clinical therapies. With the development of induced pluripotent stem cells (iPSCs), this problem has been overcome, however, current perspectives regarding clinical translation of iPSCs still remain. Unlimited differentiation potential of iPSCs, which can be used in human reproductive cloning as a risk for generation of genetically engineered human embryos and human-animal chimeras, is major ethical issue, while undesired differentiation and malignant transformation are major safety issues [2]. Accordingly, mesenchymal stromal cells (MSCs) appear as a more appropriate cell product for therapeutic purposes because of their greater biosafety profile, lower ethical challenges, as well as lower risk of tumorigenicity [3]. Due to their simple and easier isolation procedure as well as their great expansion potential, MSCs are ideal candidates for different cellular therapies [4]. The literature has well described the MSCs in their different but fundamental roles as promoters, enhancers, and playmakers of the translational regenerative medicine [5]. Although cell reconstitution is an essential component of MSC-based therapy, the therapeutic effect of MSCs is mainly a result of their potent immunomodulatory functions [6]. To optimize MSC-based therapy and achieve the appropriate therapeutic effect, several issues, such as a suitable source of MSCs, a well-characterized MSC population, the well-defined functions of MSCs, the strategy to enhance their therapeutic value, and finally the use of extracellular vesicles (EVs) as a therapeutic alternative to cellular products have to be addressed. 

MSCs are multipotent fibroblast-like cells that can be found in almost all tissues and that can differentiate into a variety of cell types. The global miRNA expression profile of MSCs varies according to the tissue of origin, species, and detection methodology, while also certain miRNAs are consistently found in all types of MSCs [7]. These miRNAs could play critical functions in MSCs by regulating several cellular properties, such as proliferation, survival, differentiation, paracrine activity, and migration. Moreover, target pathway prediction of differentially expressed miRNAs has identified different inflammation linked pathways. These recent discoveries have opened the possibility of modulating miRNAs in MSCs in order to enhance their pro-regenerative and therapeutic potential [8]. MSCs have been originally isolated from the bone marrow (BM), but during recent years, MSC-like populations have been successfully derived from other sites, including both adult and fetal human tissues [9]. Extra embryonic tissues previously seen as medical waste are increasingly recognized as a prized source of cells for therapeutic use. When compared with BM-MSCs, MSCs of neonatal origins exhibit superior proliferation ability, lower immunogenicity, and possible lower incorporated mutation—hence, they are considered as an alternative source for clinical use [10]. Among these tissues, adipose tissue (AT) and Wharton’s jelly (WJ) of the umbilical cord are actually considered as major valuable alternatives. Although these different MSC types share basic characteristics and properties, some differences in their immunological profiles could be observed. This fact suggests that the source of MSCs is important for the design of efficient and safe MSC-based immune interventions approaches [11]. WJ-MSCs were recently shown to display a distinct immunomodulatory and pro-regenerative transcriptional signature, making them interesting candidates for cell-based therapy [12]. Moreover, some limitations and inconveniences related to the diversity of isolation techniques, the impact of aging, the cell expansion rate, and the differentiation ability have prompted great interest in the study and evaluation of other tissue-derived stem cells [13]. Since their discovery in 2007, menstrual blood-derived stem cells (MenSCs) have attracted high attention because of their periodic acquisition in a non-invasive manner and the absence of moral dilemma while showing some unique features of known adult-derived stem cells. However, there is a need for a deeper characterization of their safety concern due to a variety of environmental conditions (such as epidemiological backgrounds, age, hormonal status, and pre-contraceptive) [14]. As proposed by Le Blanc, K et al., there is a need to consider every stromal cell source as an independent entity, and it is required to critically evaluate and appreciate the true phenotype of these cells and their safety when considering their use in novel cell therapies. In this connection, more attention should be paid to tissues containing cells with higher proliferative potency, broad differentiation potentials, and, most importantly, with powerful immunomodulatory effects. Preparations of MSCs are generally obtained from unfractionated tissue cells, resulting in heterogeneous cell mixtures. For tissue engineering applications, it is crucial to start with a well-defined cell population, including a well characterized cell functionality. A single marker-based selection for MSC enrichment should be more advantageous as it enables the use of a well-identified and homogeneous cell population of MSCs. Several markers may be used to selectively enrich a specific MSC population with desired functional competences [15]. The analysis and comparison of their paracrine profile identify populations with distinct phenotypes and increased functional properties, which is more interesting and safer for clinical applications than a heterogeneous population. Importantly, the choice of the cell surface marker for selecting such MSC populations depends on the source of the sample as well as the therapeutic goal to be achieved [15]. Furthermore, aldehyde dehydrogenase activity (ALDH) assay (ALDEFLUOR™) could be used to isolate and therefore characterize sub-population of MSCs. According to their ALDH activity, it is possible to distinguish and sort by fluorescence-activated cell sorting (FACS) two subsets of MSCs (referred to as ALDH+ and ALDH−). Relevant differences in gene expression related to the main properties of MSCs (proliferation, response to hypoxia, angiogenesis, phenotype, stemness, multilineage, hematopoiesis, and immunomodulation) were observed within these subsets of MSCs [16]. Regarding their definition, the International Society for Cellular Therapy (ISCT) suggested that any fibroblast-like plastic-adherent cells, regardless of the tissue from which they are isolated, should be termed mesenchymal stromal cells instead of mesenchymal stem cells, thus keeping the acronym “MSCs”. The recognized biologic properties of MSCs do not seem to meet generally accepted criteria for stem cell activity [17]. Moreover, in order to achieve uniform characterization of MSCs and facilitate the comparison of data among investigators, the ISCT as well as the International Federation for Adipose Therapeutics and Science (IFATS) have established a minimal set of standard criteria to define human MSCs according to their tissue origins [18,19]. MSCs are also defined as non-hematopoietic cells but provide the supportive microenvironmental niche for hematopoietic stem cells (HSC) and therefore promote hematopoiesis [20]. In addition to their differentiation and tissue supportive functions, MSCs have a well-established immunomodulatory function. Correct understanding of the origin and immunological properties of MSCs will help in the appropriate and safe use of the cells for clinical therapy. Accordingly, MSCs are not true immune cells but have to be considered as tissue precursor cells with immunoregulatory capacities [21]. MSCs avoid immune recognition and could display their immunomodulatory effects throughout the establishment of a tolerogenic environment, including a plethora of regulatory factors as well as distinct immune regulatory cells [22]. Surprisingly, the expression profile of these regulatory molecules as well as the underlying mechanism vary among different species. As previously discussed, there is phylogenetic distinction based on the species origin for the key molecule mediating MSC immunomodulatory effects [23]. Thus, choosing an appropriate animal model for preclinical studies of MSCs should take into account these critical observations. To achieve comparable and unambiguous results on MSC efficacy in human diseases, common and standardized protocols should be used for the immunological characterization of MSCs [24]. Importantly, MSCs and natural killer (NK) cells show cell interactions and cross modulation that impact the immunobiology of both cell types and therefore might have important consequences in the field of cell-based immunotherapy [25]. By specifically targeting and/or inducing a relevant immunoregulatory pathway, we may ensure efficient MSC therapeutic effects. Moreover, recent data highlighted that MSCs, depending on their tissue-source, present several relevant receptors, including advanced glycation end-products (RAGE) receptor; C-type lectin receptors (CLRs, including DECTIN-1, DECTIN-2, and MINCLE); leukotriene B4 (LTB4) receptors (BLT1 and BLT2); and cysteinyl leukotrienes (CysLTs) receptors (CYSLTR1 and CYSLTR2), which are potentially involved in the regulation of inflammatory and immunological responses [26]. We know that the supernatants of MSCs are full of soluble regulatory factors that play critical roles in mediating MSC immunomodulatory effects. Thus, a detailed immuno-profiling of such factors and a characterization of their therapeutic targets will be also beneficial. However, recent studies describing generation of antibodies against and immune rejection of allogeneic donor MSCs suggest that MSCs may not actually be immune privileged (hypo-immunogenic). MSCs may exert their therapeutic function through a brief “hit and run” mechanism, protecting them from immune detection. Thus, MSCs could be considered “immune evasive” through the secretion of trophic and immunomodulatory factors. Approaches that avoid allo-rejection and mitigate transplantation shock would be most useful to extend MSC persistence. Next-generation MSC therapies should be built on a foundation of thorough characterization and fine-tuning of MSC immunogenicity, survival, potency, and disease-specific mechanisms of action [27]. In their tissue residence or at sites of injury or disease, the natural and normal in vivo function of MSCs is thus as medicinal signaling cells. Their presence, their numbers, their proper activation, and their coordinated and dynamic function can have a profound impact on their therapeutic effectiveness [28]. However, recent studies have revealed that implanted cells do not survive for long and that the benefits of MSC therapy could be attributed to their paracrine activity. Secretome derivatives, such as conditioned media or exosomes, may present considerable advantages over cells for manufacturing, storage, handling, product shelf life, and their potential as a ready-to-go biologic product [29]. Thus, MSCs-derived secretome has been introduced as a promising and novel cell-free tool for regenerative medicine [30]. Upon arrival to the injured site, the communication of MSCs with the environment is an essential part of their tissue repair process. MSCs are thus considered as environmentally responsive therapeutics that can actively sense their surroundings and modulate, accordingly, their fate and behavior [31]. The vast array of bioactive factors that MSCs produce (cytokines, chemokines, growth factors) is highly sensitive to the surrounding environment (hypoxia, inflammation, infection) and is likely important in the regulation of the key biologic processes of the cell target [32]. According to their origins, tissue-derived MSCs are differentially sensitive to these environmental signals. By nature, MSCs demonstrate plasticity in their immunomodulatory effects in a way to respond to the challenges of the changing environment. The concept that MSCs can be polarized by certain stimuli provides the potential for manipulating MSCs to obtain more predictable clinical effects [33]. The nature of the stimulus received by MSCs will thus determine their effector mechanisms. Importantly, inflammation has to be considered as an important regulator of cell biology as it shapes stem cells and stemness during infection and beyond [34]. Usually, the immunomodulatory capabilities of MSCs are licensed by inflammatory cytokines. In fact, different parameters, including the concentration and type of cytokines present in the surrounding microenvironment, can influence the biological fate and response of MSCs [6]. However, such licensing should be carefully evaluated in terms of cell biology and viability. Indeed, IFNγ and TNFα synergistically triggered apoptosis of mouse BM-MSCs via the expression of inducible nitric oxide synthase (iNOS) and consequently the generation of nitric oxide (NO). NO stimulated by IFNγ/TNFα upregulated Fas expression within BM-MSCs and impaired autophagy that aggravates ER stress and promotes apoptosis [35].

The therapeutic potential of MSCs could be highly enhanced by the expression of exogenous immunological cytokines provided by transduction with viral vectors. This issue may allow enhancing MSC immuno-stimulation or immuno-suppression depending on the desired end-point of the immunomodulatory strategy but raises safety and ethical concerns [36]. Novel strategic approaches to manipulate this plasticity have to be developed to ensure efficient MSC-based therapy. Pre-conditioning of MSCs with licensing stimuli, such as natural biological compounds, may be considered in future studies [37]. In parallel, the progress of biomedical engineering, including scaffolds, biomaterials, and tissue engineering techniques, has opened new ways to overcome the low therapeutic efficacy of transplanted cells by enhancing their viability and biological activity [38]. Membrane-binding adhesive particulates significantly promoted the viability, the proliferation, and the paracrine function of adipose-derived mesenchymal stromal cells (Ad-MSCs). The production of anti-inflammatory miRNAs in exosomes of Ad-MSCs were further elevated [39]. Recently, it has been shown that newly developed synthetic biomaterial scaffolds combined with inflammatory cytokines are becoming more efficient at cell priming as they significantly improved the therapeutic potential of dental stem cells (DSC) [40].

Besides the soluble factors produced by MSCs, extracellular vesicles (EVs) have been identified as important players of this paracrine activity and thus as a part of their secretome. As a new mechanism of cell-to-cell communication, EVs could mediate the immunomodulatory effects of MSCs. By carrying different bioactive molecules (genetic materials as well as proteins), these MSC-derived vesicles might be selectively isolated and infused instead of the cells for immunotherapy purposes [41]. The release of EVs may be constitutive or consequent to cell licensing allowing thus a prompt adaptation to the environment [42]. EVs may play a major role in the therapeutic effect of MSCs and are thus considered as an attractive alternative to MSCs. However, EVs derived from MSCs are less effective than the cells themselves, and their biological effects may vary depending on the surrounding environment [43]. The EVs’ composition may be modified, suggesting that preferential packaging or exclusion of material occurs. Moreover, MSCs from different sources have been shown to have distinct EVs composition [44]. Determining the optimal strategy for isolating EVs is a critical step toward retrieving the maximal amount while ensuring the recovery of different vesicular subtypes. As recently reported, several procedures can be easily reproduced and employed regardless of the cell type used to obtain EVs [45]. The use of MSCs-derived EVs as a new therapeutic strategy for several clinical indications will open new research perspectives. Furthermore, these EVs themselves could be used as non-cellular vehicles to selectively deliver potent therapeutic factors (nanoparticles, drugs, etc.). As an alternate source of biological products, the characterization and modulation of EVs from MSCs may serve as a comprehensive basis to develop a free cell-based therapeutic strategy with enhanced value. MSC-derived exosomes induce a regulatory response in the function of B-, T-, and monocyte-derived dendritic cells. In B-lymphocytes they modulate cell function by exerting differential expression of the mRNA of relevant genes (e.g. CXCL8 (IL8) and MZB1 genes) [46]. In this context, MSC-derived EVs might be an alternative and emerging modality for several immune-based disorders and diseases [47]. The amelioration of acute graft versus host disease (aGVHD) by therapeutic infusion of BM-MSC-derived EVs was associated with the inhibition of effector T cell induction and the preservation of circulating naive T cells, probably due to their unique differentially expressed microRNAs profile [48]. In parallel, EVs derived from endometrial MSCs were highly enriched in TGFβ that contributed to their potent inhibitory effect against CD4^+^ T cell activation [49]. The preconditioning of MSCs by inflammation priming (activation with pro-inflammatory cytokines) or infection challenging (activation of the pattern recognition receptors (PRR)) and consequently their EVs could promote a better and more efficient biological response. In order to better model a more clinically relevant microenvironment, MSCs transiently exposed to a more in vivo-like culturing system, using 1% O_2_ and serum deprivation increase, showed EVs packaged with markedly higher fractions of specific protein subclasses (glycolytic, trophic and mitogenic proteins) as compared to their cells of origin, indicating regulation of their contents [50]. The promising roles of EVs as a free-cell immunoregulatory product should not underestimate their possible influence of the tumor biology (positive or negative). Furthermore, chemical or biological EVs modifications are under investigation aiming to develop more efficient anti-tumor therapies [51].

Thus, the challenges and future of cell therapy are to find an adequate and stable source of MSCs. In particular, MSCs from extra embryonic tissue have to be evaluated more as a therapeutic cell product. Once obtained, these MSCs have to be fully defined and characterized in terms of identity, functions and properties. Monitoring the disease state as well as the immunological profile of the patients will allow adequate manipulation of the regulatory machinery of MSCs. Finally, using cell-free medicinal products derived from MSCs will enhance the therapeutic value and strategy for the patients. Accordingly, all these features have to be considered and particularly developed to ensure the efficiency and safety of MSC-based therapy.
